# Prosocial Bonuses Increase Employee Satisfaction and Team Performance

**DOI:** 10.1371/journal.pone.0075509

**Published:** 2013-09-18

**Authors:** Lalin Anik, Lara B. Aknin, Michael I. Norton, Elizabeth W. Dunn, Jordi Quoidbach

**Affiliations:** 1 Marketing Department, Fuqua School of Business, Duke University, Durham, North Carolina, United States of America; 2 Department of Psychology, Simon Fraser University, Burnaby, British Columbia, Canada; 3 Marketing Department, Harvard Business School, Boston, Massachusetts, United States of America; 4 Department of Psychology, University of British Columbia, Vancouver, British Columbia, Canada; 5 Department of Economics and Business, Pompeu Fabra University, Barcelona, Spain; Middlesex University London, United Kingdom

## Abstract

In three field studies, we explore the impact of providing employees and teammates with *prosocial bonuses*, a novel type of bonus spent on others rather than on oneself. In Experiment 1, we show that prosocial bonuses in the form of donations to charity lead to happier and more satisfied employees at an Australian bank. In Experiments 2a and 2b, we show that prosocial bonuses in the form of expenditures on teammates lead to better performance in both sports teams in Canada and pharmaceutical sales teams in Belgium. These results suggest that a minor adjustment to employee bonuses – shifting the focus from the self to others – can produce measurable benefits for employees and organizations.

## Introduction

A recent survey revealed that just 46% of Americans are satisfied with their jobs, the lowest level recorded by the Conference Board [1] in the past two decades. Yet over the same time frame, Americans have come to spend more and more of their time at work [2]. Taken together, this trend suggests that employees are becoming more and more unhappy more and more of the time at work, hardly a formula for a healthy and productive workplace. In this increasingly negative environment, how can employers incentivize their employees to increase their happiness, job satisfaction, and ultimately their job performance?

Certainly, designing effective incentive schemes is a central challenge for a wide range of organizations, from multi-national corporations to academic departments. In pursuit of identifying the most effective strategies, organizations have devised an impressive variety of such bonuses, from fixed salaries to pay-per-performance, from commissions to end-of-year bonuses. We suggest that the wide variety in such schemes masks a shared assumption: That the best way to motivate employees is to reward them with money that they then spend on themselves. We propose an alternative means of incentivizing employees – what we term “prosocial bonuses” – in which organizations provide employees with bonuses used to engage in prosocial actions towards charities and co-workers.

Below, we first review research exploring existing methods of increasing workplace performance, including individual-based and team-based bonus schemes, which tend to reveal both benefits and unexpected costs. We then briefly review the literature on the benefits of improving social life in the work place, such as increasing employee citizenship behaviors. Next, we argue that prosocial bonuses mitigate some of the issues that arise with individual- and team-based compensation schemes, while retaining the benefits of improving employee’s social lives in the workplace. Finally, we examine the impact of these prosocial bonuses on employee satisfaction and team performance, by reporting results from three “proof of concept” field experiments conducted in different countries.

### Individual- and Team-Based Incentive Schemes

When asked why they work, individuals most commonly reply “money” [3]. But what is the effect of money on employees’ job satisfaction and performance? On one hand, monetary bonuses have been found to produce positive effects – increased productivity, effort, performance, and job satisfaction [4]–[9]. Individual bonuses increase job satisfaction in part because employees see their time and effort being rewarded [10]–[13]. From pay-per-performance to piece rate compensation schemes to profit sharing to bonuses, individual-based incentive schemes can lead to improved employee outcomes [8], [14]–[18].

On the other hand, individual incentives – such as large bonuses – are often surprisingly ineffective in increasing employee morale and productivity [19]–[20]. Rewarding individual employees can produce negative outcomes by eroding workplace cohesion [21], as employees become reluctant to share information with others even at the expense of reduced output [22]. Relative comparisons at the individual level create competition which results in decreased trust, sharing and teamwork [23]–[25]; in Drago and Turnbull [26], for example, tournament-based compensation led to decreased helping behavior and increased the potential for sabotaging other workers.

In an effort to prevent such negative competitive dynamics that can result from individual-based bonuses, organizations often turn to incentivizing employees for their collective performance, encouraging cooperation and teamwork rather than competition [27]–[29]. Indeed, a growing body of research suggests that interpersonal relationships enable employees to experience their work as important and meaningful [30]–[36]. Furthermore, evidence suggests that interpersonal relationships often enhance employees’ motivations, opportunities, and resources at work [37]–[40]. Positive interpersonal relationships with coworkers provide social support and a buffer from stressful events [41]–[43], which in turn predict team commitment [44], job engagement [45]–[46], and job satisfaction [47]–[49].

In some cases, team-based compensation schemes have been shown to raise this sense of cooperation and cohesiveness between team members [22], [50], inducing them to exert additional effort toward helping one another [51]–[54]. Importantly, such increased cooperation due to interdependent rewards has been shown to improve team performance [55], suggesting that team-based bonuses may be an effective means of improving employee social life. As with individual-based bonuses, however, team-based bonuses offer important advantages but also potential drawbacks – such as free riding [56], motivational loss due to the perception of inequity [57], and suboptimization of team goals [58]. Thus while team-based bonuses have the potential to improve relationships between co-workers, they can also lead to “antisocial” behaviors – and decreased employee outcomes.

### Prosocial Bonuses

We suggest that prosocial bonuses offer an alternative approach that has the potential to provide some of the same benefits as team-based compensation – increased social support, cohesion, and performance – while carrying fewer drawbacks. Research suggests that the desire to help others is a need deeply rooted in human nature [59]–[60], and that giving to others has a causal impact on increasing happiness and life satisfaction [61]–[62]. At the organizational level, previous correlational research suggests that prosocial behavior in the workplace – often termed citizenship behaviors – is linked to employee morale and performance [63]: the extent to which employees perceive themselves and their organizations as prosocial predicts organizational commitment [64]–[66]. We suggest that prosocial bonuses can have a *causal* impact on employee satisfaction and performance, such that providing employees with money to help others would have a greater organizational impact than providing employees with money to spend on themselves.

We note that we are not the first researchers to examine the interplay of incentives and prosocial behavior; indeed, several investigations point to the potential risk in mixing money with altruism [67]: paying children to collect money for charity decreases their efforts [68], publicly rewarding adults for earning money for charity also decreases effort [69], and paying friends to help with a move reduces the amount of help received [70]. Unlike these kinds of “prosocial incentives,” however, the prosocial bonuses we provide in the experiments below are not contingent upon or linked to any behavior – employees are simply given money by the firm to spend prosocially. In this sense, our investigation uses a version of a “reciprocity by proxy” strategy outlined by Goldstein, Griskevicius, and Cialdini [71]. In this investigation, guests who were informed that a hotel had already given a donation to an environmental cause were more likely to reuse their towels than those who were told the hotel would make a donation *only if* they reused their towels; their results showed that providing the prosocial bonus up front was more effective than linking the incentive directly to the behavior. Following this logic, we predicted that offering employees prosocial bonuses that were not linked to any current behavior or expectation of future behavior would be effective in increasing employee satisfaction.

### Overview of the Present Research

We examine whether randomly assigning employees to engage in prosocial behavior – via prosocial bonuses – can have a causal impact on employee well-being, job satisfaction, and job performance. In our field studies, some employees and teammates are given non-contingent “prosocial bonuses” – money that they receive as a windfall that they are encouraged to spend in a prosocial manner. In Experiment 1, we give some employees of a company the opportunity to donate money to charity, examining the impact of this intervention on both employee well-being and job satisfaction. In Experiments 2a and 2b, we move beyond assessment of psychological constructs to behavioral measures; by comparing prosocial versus personal bonuses, we investigate their impact on team performance in the two different contexts of sales teams and sports teams.

## Materials and Methods

### Ethics Statement

Data collection for Experiment 1 was approved by the Harvard University Behavioral Research Ethics Board. Data collection for Experiment 2a was approved by the University of British Columbia’s Behavioral Research Ethics Board (B06-0557). Data collection for Experiment 2b was overseen by University of Liège. Written informed consent was obtained for all studies.

### Experiment 1

In Experiment 1, we examine the impact of prosocial bonuses on the most widely studied attitude in the field of organizational behavior, job satisfaction – broadly defined, employees’ subjective evaluation of their work experience [72]–[73]. The large number of investigations examining factors that influence job satisfaction have tended to focus on two fundamental determinants: (1) aspects of employees, such as individual differences in self-esteem or education [74]–[78] and (2) aspects of the job itself, such as communicating clear task goals and giving feedback when those goals are achieved [79]–[84]. Adding a novel contribution to the literature on job satisfaction, we examine the impact of prosocial bonuses. To do so, we assigned some employees of a large bank to receive a prosocial bonus in the form of money from the company to donate to charity, and examined the impact of spending this bonus on job satisfaction, compared to employees not given this bonus.

#### Participants

A total of 300 employees at an Australian bank were invited by their employer to participate in an experiment; 121 of these employees did not respond to the initial email and were therefore not included in our sample. Of the 179 employees that did respond to the invitation, 46 employees completed only the Time 1 survey in which they reported their age, gender, salary, years at company as well as their happiness and job satisfaction. These 46 participants did not differ from our main sample in terms of age, gender, income or years at company, Time 1 happiness or job satisfaction (*ts*<1.13, *ps* >.26). Employees completing only the Time 1 survey were not included in the analyses below, leaving a final sample of 133 bank employees (59 percent female; *N*_control_ = 48, *N*_$25_ = 41, and *N*_$50_ = 44) with a wide range of income, age, and years at the company (Table 1).

**Table 1:** Employee demographics (Experiment 1).

Age (years)	%	Income ($AUS)	%	Years at Company	%
21–29	23.3	$20,001–$50,000	10	<1	14
30–39	38.3	$50,001–$100,000	42	1–2	18
40–49	26.3	$100,001– $150,000	34	3–5	21
50–59	12	$150,001 – $200,000	11	6–10	12
		$200,001 – $500,000	3	11–15	12
				>15	23

#### Design and procedure

On November 17, 2008, all employees received an email from their employer asking them to participate in a multi-stage experiment on workplace attitudes. Employees were assured that their participation was voluntary and that their responses would be anonymous. If employees followed a link indicating their willingness to participate, they were directed to the Time 1 survey. On the Time 1 survey, participants reported their gender, age, and salary. Because this was a field experiment conducted during a work day, we asked participants to complete single-item measures of happiness and job satisfaction at Time 1 and Time 2. Participants rated how happy they felt on the 5-point scale (1: *very slightly or not at all* to 5: *extremely*) used in the Positive and Negative Affect Schedule [85]. This single-item measure has been previously shown to be highly correlated with the full scale (*r* = .48, *p*<.001) [86], and similar single-item measures of happiness have been widely used in the well-being literature [87]–[88]. To assess job satisfaction, participants completed a measure drawn from the Michigan Organizational Assessment Questionnaire, rating their agreement with the statement “All in all I'm satisfied with my job” on a 7-point scale (1: *strongly disagree* to 7: *strongly agree;* 89). Single-item measures of job satisfaction have been shown to correlate with longer assessments, and yield adequate validity [90]–[92].

Two weeks later, on December 3, 2008, based on random assignment, employees in the control condition were sent an email that directed them to complete the Time 2 survey. Employees randomly assigned to one of the two experimental conditions were informed that the company had given them a charity voucher worth approximately $25 or $50 US at the time to donate to a charity of their choice. Participants in the two charity voucher conditions followed a link that took them to a charity website (KarmaCurrency.com.au) where they could donate to a wide range of charities of their choice. After completing the donation, participants were automatically redirected to the Time 2 survey. Voucher redemption data shows that about half of the employees redeemed their charity vouchers on the day they received it (December 3, 2008). The remaining vouchers were redeemed during the following two weeks with the last redemption on December 18, 2008.

### Experiments 2a & 2b

Experiment 1 revealed that providing employees with the opportunity to spend prosocial bonuses can yield two psychological benefits: increased happiness and job satisfaction. Indeed, employees who donated $50 to charity on behalf of their company reported increased happiness and job satisfaction. Do the benefits of prosocial bonuses extend beyond employee well-being to improving actual performance – and the organizations’ bottom line? As with job satisfaction, previous research has focused on two categories of predictors of job performance, some examining the links between employees’ individual differences (e.g., their general aptitude or conscientiousness) and their performance, and other research examining how aspects of the job itself can improve or undermine performance [77], [93]–[96]. We suggest that prosocial bonuses offer an additional approach to impacting job performance; we expected that compared to personal bonuses, prosocial bonuses would have a larger impact on job performance.

In addition to documenting the impact of prosocial bonuses on team performance, we also widened our investigation in three ways. First, we sought to extend the time course of our experiment to examine the longer-term effects of prosocial bonuses. In Experiment 1, we measured job satisfaction immediately after the prosocial bonus, which we acknowledge is likely when the impact of giving was at its greatest. We assess more delayed or extended benefits of prosocial bonuses in Experiments 2a and 2b. Second, we explored the impact of a different form of prosocial bonuses; to do so, we redirected generous spending from external charitable causes to co-workers and teammates within the organization. Third, Experiment 1 compared the effects of prosocial bonuses to a control condition; in Experiments 2a and 2b we directly compared the impact of prosocial and personal bonuses, by giving members of some teams money to spend on their teammates and members of other teams money to spend on themselves. Due to logistical reasons, a control condition could not be included in Experiments 2a and 2b.

### Experiment 2a: Sports Teams Methods

#### Participants

Sixty-two students (83 percent male; *M*_age_ = 20.49, *SD* = 2.6) on 11 recreational dodge ball teams (*M*_members_ = 4.71, *SD* = 1.4) completed the experiment at the University of British Columbia for a chance to win $100. Potential participants were informed that one person would be selected to win the $100 cash prize.

#### Procedure

Teams were approached in person by a research assistant in a recreation center on campus and invited to participate in a study. Members of participating teams completed a basic demographics survey in which they noted their age, gender, annual income and student status. Each team was randomly assigned to the personal or prosocial bonuses condition. Within each team, approximately one-third of team members were randomly selected to receive $20 USD (∼$20 CDN) to spend over the subsequent week. Participants in the personal bonus condition were instructed to “spend the money on a bill, expense, or gift for yourself”, while participants in the prosocial bonus condition were instructed to “spend the money on a teammate” who was randomly selected. Both personal and prosocial spending instructions were presented in written form and then explained by a research assistant to ensure participants understood the instructions.

#### Team performance

Performance was assessed with the percentage of games won out of total games played on the date of the initial survey (Time 1) and approximately two weeks later (Time 2). Only team level performance could be measured, as individual players’ statistics were not collected by the recreational dodge ball league.

### Experiment 2b: Sales Teams Methods

#### Participants

One hundred and twelve salespersons at a Belgian pharmaceutical company were emailed by their Human Resources Department with an invitation to take part in an experiment. All of the salespersons indicated willingness to participate and provided their demographic information. Twenty-four salespeople were excluded from the experiment for various reasons. Specifically, for ten salespersons we could not get performance data from the company. Some salespersons, for example, were active in two different sales territories, sharing their sales performance with multiple teams. Others were in charge of special projects for which we could not have access to a performance indicator. An additional fourteen salespersons who were team leaders were excluded as we wanted to examine giving among peers, rather than between employees and supervisors. The remaining 88 salespersons (50 percent male; *M_age_* = 36.0, *SD* = 6.9) working in 14 teams (*M_members_* = 6.3, *SD* = 3.0) completed this experiment in exchange for a chance to win an iPod. Participants were assured that participation was voluntary and their responses would remain confidential.

#### Design and Procedure

The pharmaceutical salespersons worked in teams that were in charge of the same geographical region. Although each salesperson worked alone, team members would share strategic information about prospects (e.g., “You should go to that business because the owner doesn’t like me”). Each sales team was randomly assigned to the prosocial or personal bonuses condition. Because teams varied in size, we randomly selected approximately one-third of team members, and at a companywide event two weeks after the initial email, we gave these individuals $22 USD (15 Euros) to spend by the end of the week. Participants were informed that the funds were provided as part of a study conducted by independent researchers. On personal bonus teams, participants who received money were asked to “spend it on a bill, expense, or gift for yourself” (as in [62]), whereas on prosocial bonus teams, participants who received money were instructed to “spend it on a teammate” who was randomly selected from the remaining team members and specified for each spender. All participants receiving funds to spend were asked to complete the spending by the end of the week. While one-third of the salespersons were assigned to be spenders (i.e. someone given money to spend on themselves or a coworker), the remaining participants were assigned as receivers (i.e. someone who received a gift from a coworker) or third-party observers (i.e. someone not assigned as a spender or receiver). Receivers were not informed that they would receive a gift from a co-worker. In order to avoid confusion at the companywide event, participants assigned to be receivers and third-party observers also received envelopes with a brief note thanking them for their participation in the study but they were not informed of the spending manipulation. Everyone was instructed to open the envelopes alone at home.

At the end of the week, spenders reported how they had used the money, and receivers were reported whether they had received any gifts, enabling us to confirm spenders’ reports. The twenty-four salespersons excluded from our study were not eligible to be spenders or receivers. We provided the company with the funds to be distributed to the salespersons, who were fully informed that the study was conducted only by independent researchers, and that the company would not have access to any of the data.

#### Team performance

Performance was assessed immediately before (Time 1) and one month after our spending intervention (Time 2). Pharmaceutical salespeople promote their product to physicians, pharmacies, and hospitals, rather than selling directly to customers. As such, the standard indicator of pharmaceutical sales team success is the total monthly sales collected by each pharmaceutical sales team (in Euros) in the geographical region under their purview. Therefore, we used monthly team sales as our measure of team performance.

## Results

### Experiment 1

#### Happiness

A preliminary ANOVA confirmed that there was no difference between conditions in Time 1 happiness, *F*(2, 130)  = .12, *p*>.85, *η_p_* ^2^ = .02; we therefore entered experimental condition into an ANCOVA predicting Time 2 happiness, controlling for Time 1 happiness. We observed a significant main effect of condition, *F*(2, 129)  = 5.85, *p*<.005, *η_p_* ^2^ = .08. Follow-up analyses showed that participants who received a $50 USD charity voucher reported being significantly happier, *t*(43)  = 5.12, *p*<.001, whereas happiness levels were unchanged from Time 1 to Time 2 for those in the control and $25 USD conditions, *t*s <1 (Table 2).

**Table 2:** Change in happiness and job satisfaction between Time 1 and Time 2 as a function of condition (Experiment 1).

	Time 1		Time 2	
	Happiness	Job Satisfaction	Happiness	Job Satisfaction
Control Condition (*N* = 48)	3.48 (.83)	5.15 (1.50)	3.56 (.80)	5.25 (1.35)
$25 USD Condition (*N* = 41)	3.56 (.87)	5.37 (1.61)	3.51 (.95)	5.12 (1.35)
$50 USD Condition (*N* = 44)	3.52 (.70)	5.23 (1.29)	3.98 (.51)	5.55 (1.07)

#### Job Satisfaction

As with happiness, a preliminary ANOVA confirmed that there were no between-group differences in Time 1 job satisfaction, *F*(2, 130)  = .54, *p*>.77, *η_p_* ^2^ = .004. Entering condition into an ANCOVA predicting Time 2 job satisfaction, controlling for Time 1 job satisfaction, revealed a significant main effect of condition, *F*(2, 129)  = 3.14, *p*<.05, *η_p_* ^2^ = .05. As with happiness, participants who received a $50 USD charity voucher showed an increase in job satisfaction, *t*(43)  = 2.46, *p*<.02, which was unchanged for those in the control and $25 USD conditions, *t*s<1.19 (Table 2).

### Experiment 2a

#### Spending examples

Participants who received a personal or prosocial bonus were asked to report how they spent this money. On personal bonus teams, spenders reported buying items for themselves such as sportswear, small jewelry, CDs, food, and alcohol. On prosocial bonus teams, spenders reported buying items for others such as books, wine, a plant, a stuffed animal, a piñata and paying a teammate’s sports league fee.

#### Spending condition and team performance

To confirm that there were no significant differences in initial performance, we entered condition (personal bonus vs. prosocial bonus) into an ANOVA predicting Time 1 performance; this analysis revealed no significant effect, *F*(1, 10)  = .10, *p* = .77. As in Experiment 1, therefore, we entered the same variables into an ANCOVA predicting Time 2 performance, controlling for Time 1 performance. We found a marginal main effect in the predicted direction, whereby prosocial bonus teams performed better than personal bonus teams, *F*(1, 8)  = 3.75, *p* = .09, *η*_p_^2^ = .32 (Table 3).

**Table 3:** Change in sports and sales team performance between Time 1 and Time 2 as a function of condition (Experiments 2a and 2b).

	Time 1		Time 2	
	Sport Teams	Sales Teams	Sport Teams	Sales Teams
	*Percentage of*	*Sales in*	*Percentage of*	*Sales in*
	*Games Won*	*Dollars*	*Games Won*	*Dollars*
Personal Bonuses	50% (35%)	5761 (3312)	43% (44%)	5776 (3508)
Prosocial Bonuses	50% (55%)	4892 (3184)	81% (31%)	5170 (3343)

Next, we examined the impact of prosocial vs. personal bonuses on the change in performance from Time 1 to Time 2. In the prosocial bonuses condition, sports teams showed a large, but statistically marginally significant increase in performance, *t*(5) = 1.87, *p* = .12, *d* = .76. Meanwhile, in the personal bonuses condition, there was no evidence for improved performance, *t*(4)  = 0.39, *p* = .72, *d* = .17 (Table 3).

Another way to demonstrate the effectiveness of these interventions is to calculate the return on investment for prosocial and personal bonuses. On sports teams, every $10 people spent on themselves led to a two percent decrease in winning percentage, whereas every $10 spent prosocially led to an 11% increase in winning percentage.

### Experiment 2b

#### Spending examples

The salespeople who received a personal or prosocial bonus were asked to report how they spent the allotted funds. On personal bonus teams, spenders reported buying items for themselves such as food, alcohol and groceries. On prosocial bonus teams, spenders reported buying items for others such as gift card, chocolate, wine, and treating a teammate to lunch.

#### Spending condition and team performance

As in Experiment 2a, to confirm that there were no significant differences in initial performance, we entered condition (personal bonus vs. prosocial bonus) into an ANOVA predicting Time 1 performance; this analysis revealed no significant effect, *F*(1, 12)  = .24, *p* = .63. Therefore, we entered the same variables into an ANCOVA predicting Time 2 performance, controlling for Time 1 performance. As in Experiment 2b, we found a marginal main effect, whereby prosocial bonus teams performed better than personal bonus teams, *F*(1, 11)  = 2.31, *p* = .16, *η*_p_^2^ = .17 (Table 3).

Although the simple effect should be interpreted with caution given the very small sample size, closer examination suggests that prosocial bonuses were effective in improving performance from Time 1 to Time 2. That is, in the prosocial bonuses condition, sales teams showed a large and significant increase in performance from Time 1 to Time 2, *t*(6)  = 2.70, *p*<.04, *d* = 1.02. Meanwhile, in the personal bonuses condition, there was no evidence for a performance improvement, *t*(6)  = 0.10, *p* = .92, *d* = .04 (Table 3).

Once again, it is possible to conceptualize the effectiveness of these interventions by calculating the return on investment for prosocial and personal bonuses. On sales teams, for every $10 USD given to a team member to spend on herself, the firm gets just $3 USD back – a net loss; because sales do not increase with personal bonuses, personal bonuses are wasted money. In sharp contrast, for every $10 USD given to a team member to spend prosocially, the firm reaps $52 USD.

The results of Experiments 2a and 2b are similar; teams that received prosocial bonuses outperformed teams that were given personal bonuses. These results emerged despite the logistical and statistical limitations of samples of team data. Indeed, the small sample size may explain why the effects are marginal in both experiments.

Therefore, to more accurately estimate the true effect size of prosocial bonuses on performance, we conducted a meta-analysis. Meta-analyses are frequently used to combine the results of two or more studies, allowing researchers to arrive at more accurate conclusions than can be presented in a single study [97]–[100]. This method is advantageous when several experiments favor the same result but fail to reach significance due to small sample size [101].

Taking this approach with our data, across Experiments 2a and 2b, we combined the effect sizes for the change from Time 1 to Time 2 performance in prosocial and personal teams. The meta-analysis revealed that prosocial teams performed significantly better from Time 1 to Time 2 as revealed by the significant *Z* = 2.66, 95% CI (.31, 2.02). We repeated the same analysis for the change in performance from Time 1 to Time 2 for personal teams, which revealed a nonsignificant Z = .03, 95% CI (-.67,.88). These results from the meta-analysis show that the change in performance from pre- to post-bonuses was significant in prosocial teams while not significant in personal teams.

## Discussion

We offer initial evidence of the causal impact of increasing prosocial behavior via the provision of prosocial bonuses to employees at an Australian bank, members of dodge ball teams in Canada, and pharmaceutical salespeople in Belgium. Taken together, our studies show that when organizations give employees the opportunity to spend money on others – whether their co-workers or those in need – both the employees and the company can benefit, with increased happiness and job satisfaction and even improved team performance. Specifically, in Experiment 1, employees who had the opportunity to make a substantial donation to charity ($50 USD) on behalf of their company reported enhanced happiness and job satisfaction in the short term, compared to those in the control condition. In Experiments 2a and 2b, we extended these findings to team performance in the longer term, showing that teams performed better when participants were assigned to spend money on their fellow team members than when given a more standard bonus: money to spend on themselves. Across the studies, we show that prosocial bonuses can benefit both individuals and teams, on both psychological and “bottom line” indicators, in both the short and long-term. Unlike some research suggesting a weak link between factors that improve job satisfaction and those that improve job performance [102]–[104] our results suggest that prosocial bonuses have a meaningful impact on both metrics.

How might prosocial bonuses lead to increased happiness, job satisfaction and team performance? Because our studies were conducted in the field, we were unable to conduct extensive surveys assessing likely mediators of the impact of prosocial bonuses. While the beneficial impact of prosocial spending on happiness is well-established [62], [86], a key goal for future research is to explore underlying mechanisms of the prosocial bonus-performance link, with several clear possibilities worthy of investigation. First, prosocial bonuses may lead to the strengthening of existing relationships and even the formation of new relationships; such positive interpersonal relationships predict job engagement [45], [46] and job satisfaction [47]–[49]. Second, and relatedly, prosocial bonuses might lead to increased cooperation and cohesiveness between team members, which can improve team performance in part by encouraging helping behaviors [51]–[55]. Finally, prosocial spending may increase general feelings of reciprocity among members of organizations, leading both to greater cooperation and punishment of “shirkers” or “free riders” – those employees who are not contributing to the goals of the organization [105]–[110].

Along similar lines, future work should examine whether the impact of prosocial bonuses on team performance is driven by actions of the spenders, receivers, or a combination of the two. Since we were not able to measure individual performance in sales and sports teams, we could not pinpoint whether prosocial bonuses increased team performance by motivating individual-level contributions or team-level operations. Assessing individual level contributions would also allow researchers to examine how additional team members -- who were neither spenders nor receivers -- respond to this type of intervention. Future experiments that include both prosocial and personal bonuses while assessing these – and other – constructs will add to our understanding of the benefits of prosocial bonuses.

We note that Experiment 1 included a prosocial bonus condition and a control condition but not a personal bonus condition, whereas Experiments 2a and 2b included prosocial and personal bonus conditions but not a control condition; in addition, Experiment 1 included two levels of bonuses, whereas in Experiments 2a and 2b the bonus amount was kept constant. These decisions were driven by logistics. Our study sites were not interested in including a personal bonus in Experiment 1 but did allow us to include two levels of prosocial bonus; they were interested in including both personal and prosocial bonuses of a fixed amount but not a control condition in Experiments 2a and 2b. Of clear interest for future research is more systematic and comprehensive variation of all of these factors, crossing many bonus levels with both personal and prosocial bonuses. In addition, as we noted in Experiment 2, our observations at the team level are low in number (150 participants become just 25 teams across Experiments 2a and 2b); scaled-up experiments that utilized more teams would also build on the “proof of concept” experiments we present here.

It would be particularly interesting to examine employees’ sensitivity to bonus levels as a function of whether those bonuses are personal or prosocial. Receiving $10 or $20 for oneself is likely to lead only to the purchase of one or two additional coffees, and therefore seems unlikely to impact employee satisfaction or job performance. Buying a $20 gift for a coworker instead of a $10 gift, on the other hand, may encourage people to be even more creative and thoughtful in their gift choice, making the experience more impactful for both the giver and the receiver – and possibly leading to a bigger return on investment for the organization. More broadly, a $10 personal bonus from one’s organization may seem like a trifling or insufficient reward, leading to a decrease in motivation [71] – “I worked all year and they only gave me $10?” – whereas our results suggest that the same small sum of money spent prosocially has a markedly different, and positive, effect.

Related to the above, $25 USD was not sufficient to increase employee satisfaction in Experiment 1, but the meta-analysis for Experiments 2a and 2b suggests that $20 USD may be able to increase team performance. We suggest that this difference is likely due to the different form that prosocial bonuses took in the two studies. Recent research suggests that face-to-face giving has a larger impact on happiness than giving at a distance: not only are people more likely to donate money to toward single individuals than to larger organizations [111]–[112], but the closer the link between giver and receiver, the bigger the happiness benefits: people who give money to others are happier when they give face-to-face rather than remotely, and spending money on close friends leads to more happiness than spending on more distant acquaintances [113]–[114]. As a result, it is not surprising that the same amount of money (∼$20 USD) goes further in Experiments 2a and 2b than in Experiment 1, given the social nature of the team expenditure compared to the relatively impersonal donation to charity. Perhaps even more importantly, whereas in Experiment 1 employees were givers only, in Experiments 2a and 2b teammates were both givers *and* receivers: for every salesperson who gave a gift, there was a salesperson who received that gift, likely another contributor to the greater impact of prosocial bonuses in Experiments 2a and 2b. Importantly, the observed boost in employee satisfaction and happiness only for the $50 USD and not for the $25 USD in Study 1 helps rule out the possibility that our results are simply due to demand effects. Demand effects should have influenced both of the prosocial donation conditions (e.g., $25 USD and $50 USD) equally. Thus, if employees felt that they should be happy after giving, then the boost in happiness would have been observed across all prosocial spenders, not just for employees who gave $50.

Our experiments provide preliminary evidence for the potential utility of prosocial bonuses, though future research is needed. Given that existing incentive schemes have important drawbacks, it is worthwhile to consider creative new approaches to incentivizing employees. That said, we assume that prosocial bonuses may have drawbacks of their own, which future research should document. In particular, it seems likely that prosocial bonuses could backfire if they were introduced by companies as a *replacement* for more standard bonuses. Because many companies already allocate funds for charitable giving and employee entertainment, however, it may be possible for companies to reap the benefits of prosocial bonuses by providing some of these existing funds directly to employees, who can then use this money to make donations to charity or to benefit co-workers—potentially increasing job satisfaction and performance in the process. Relatedly, prosocial bonuses were unconditional in our experiments; future research could examine whether bonuses conditional on performance or based on competition would prove as effective in increasing job satisfaction and performance.

We opened by noting that recent surveys indicate that job satisfaction is at a twenty-year low in the United States even as Americans have come to spend more and more of their time at work. This additional time at work, of course, often comes at the expense of devoting time to pursuits known to be linked to well-being, from forming social connections to engaging in prosocial acts such as volunteering [2], [115]–[116]. We suggest that rather than force employees to make a losing tradeoff between social life and work life, employers can focus instead on using prosocial bonuses to create a more altruistic, satisfying, and productive workplace.
